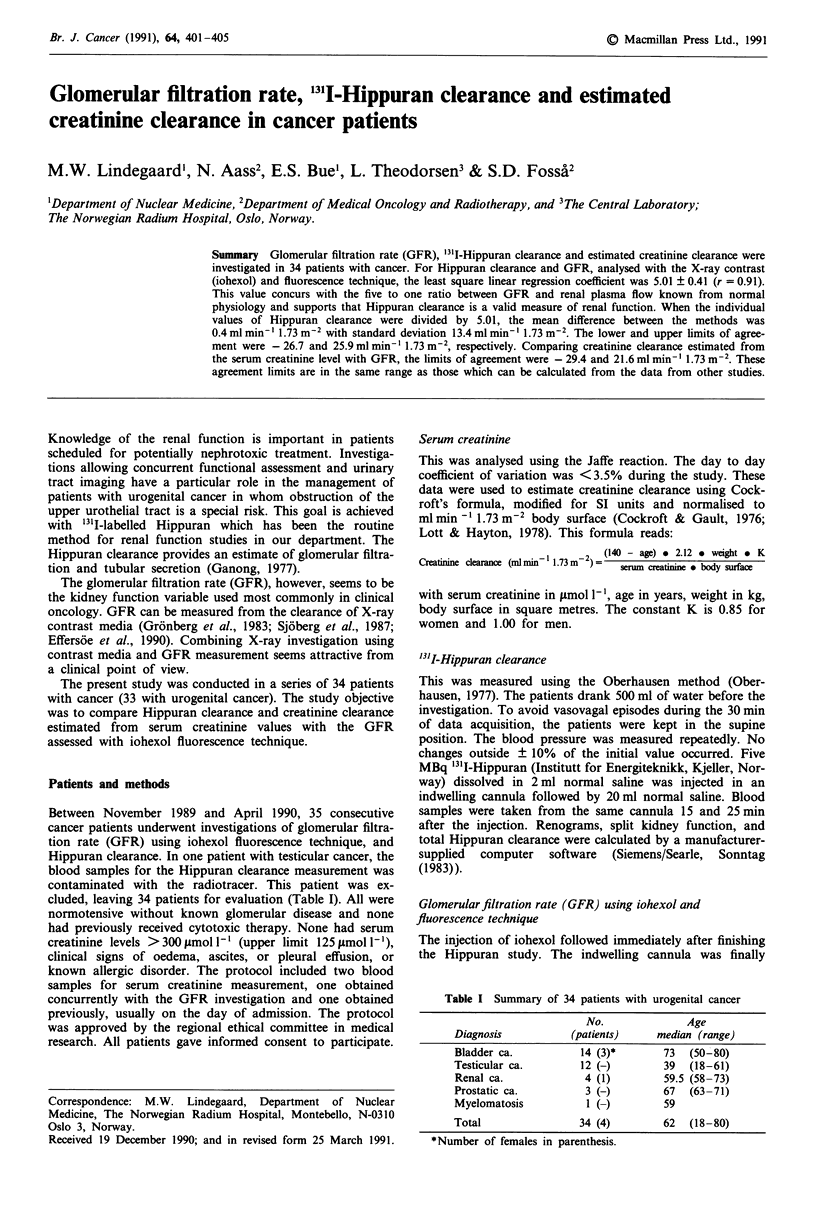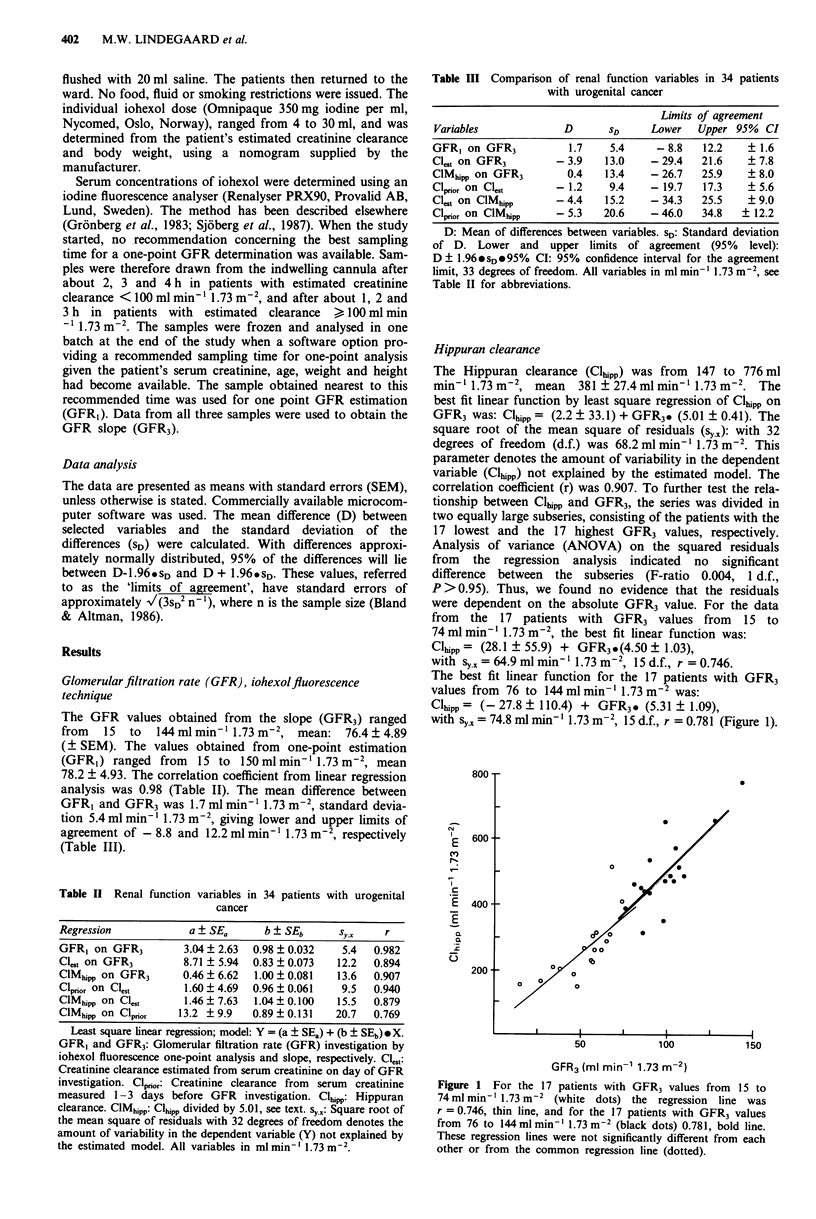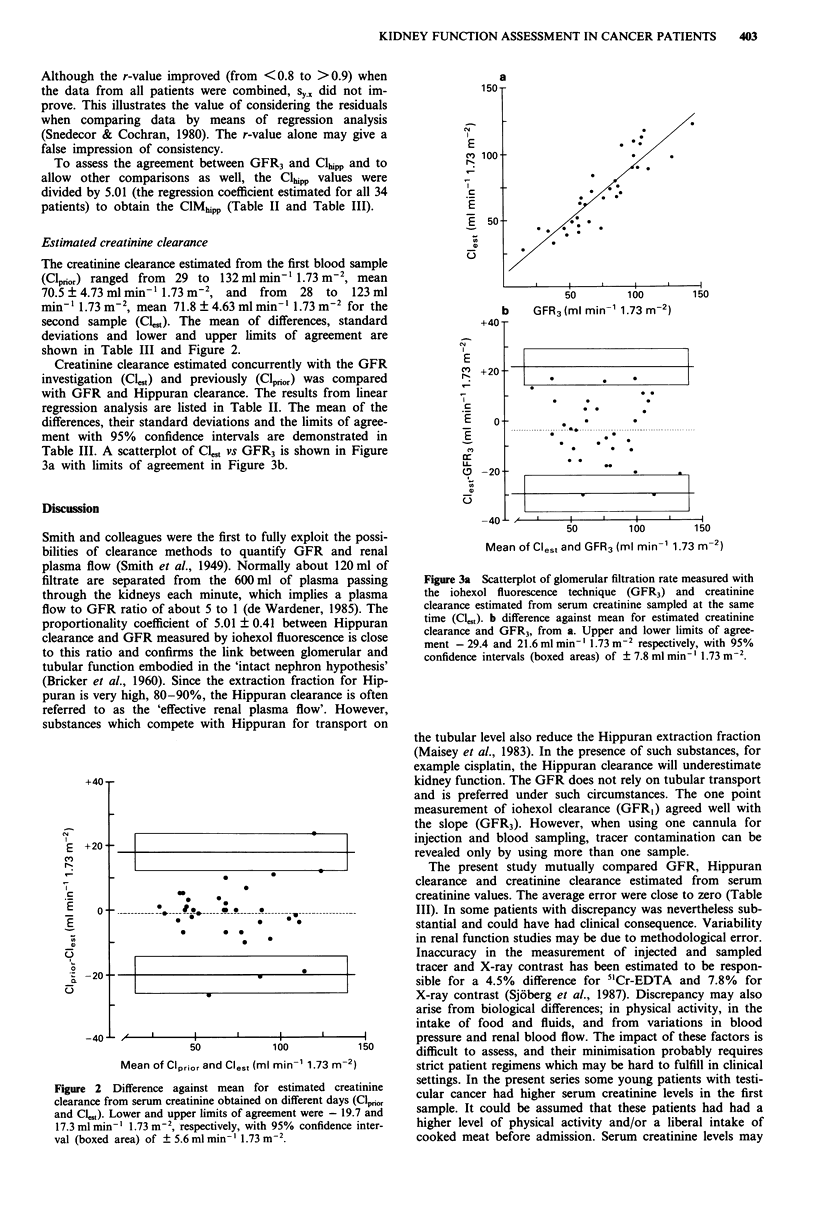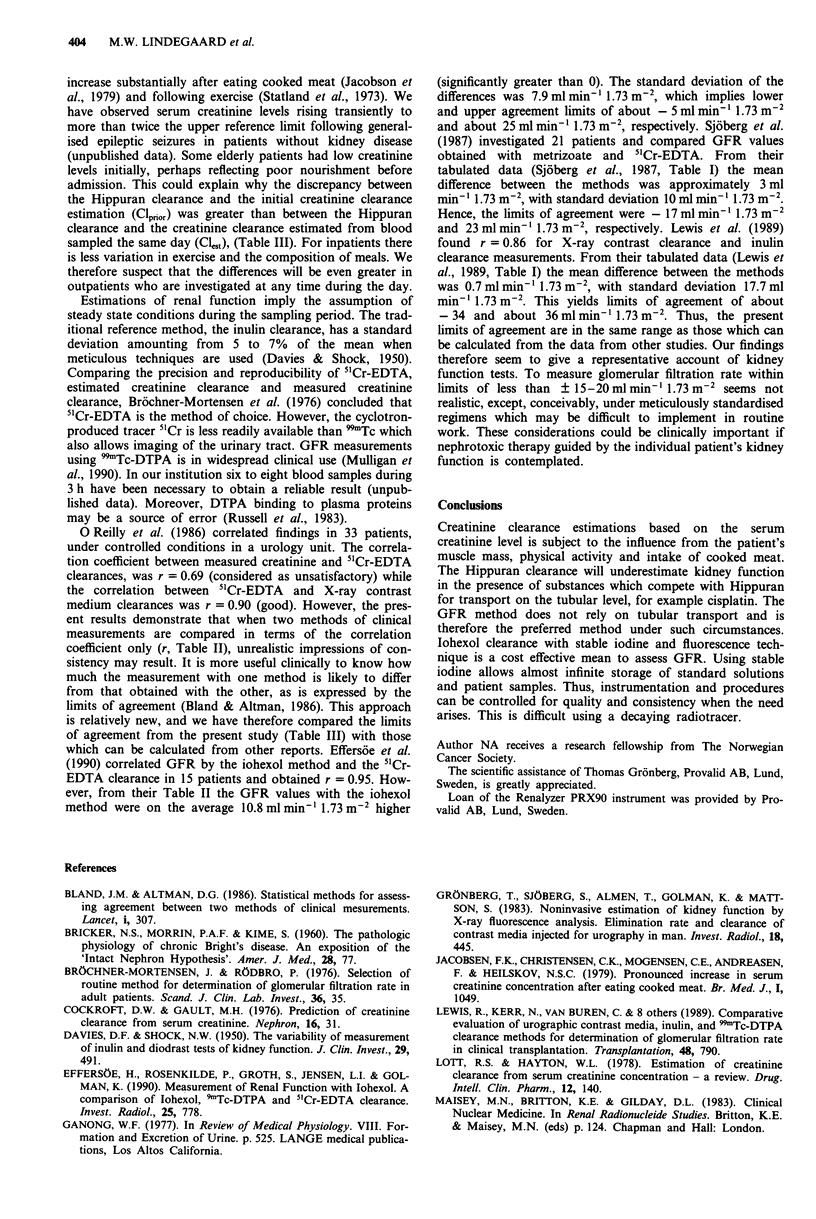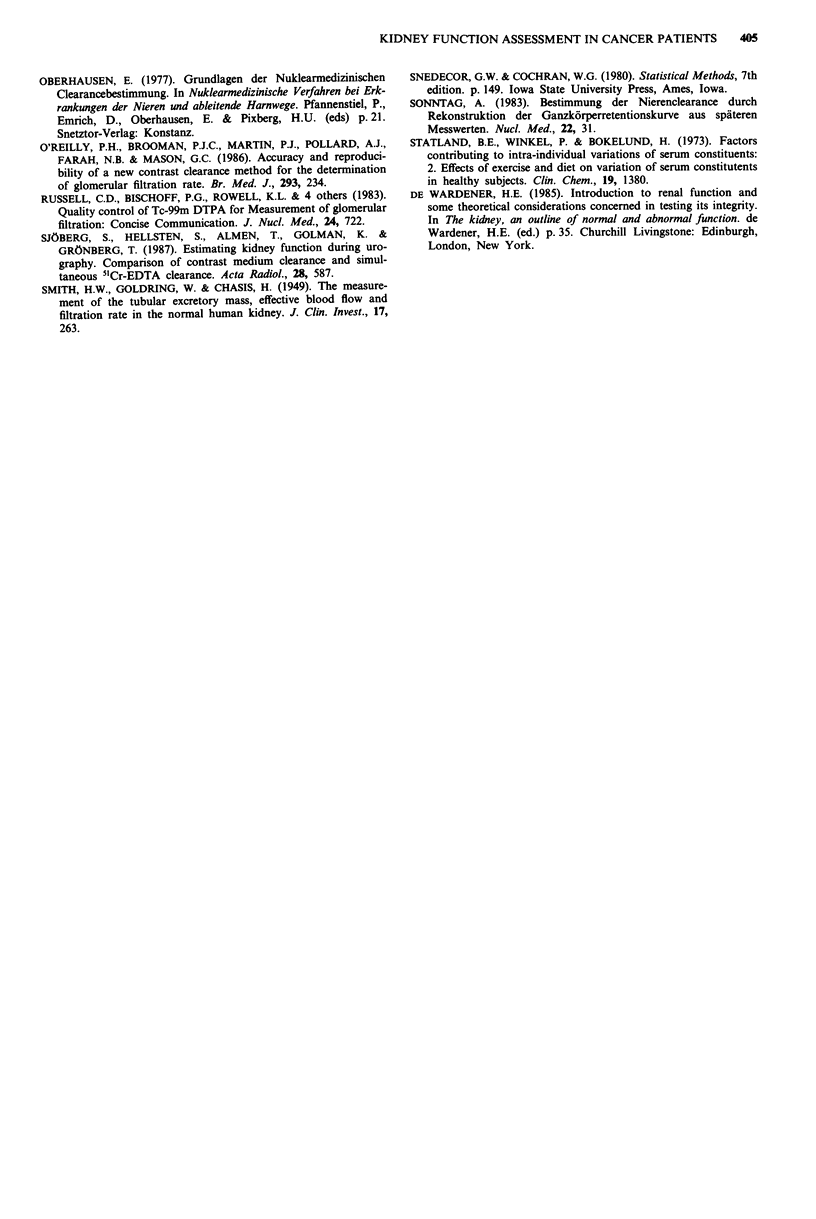# Glomerular filtration rate, 131I-hippuran clearance and estimated creatinine clearance in cancer patients.

**DOI:** 10.1038/bjc.1991.317

**Published:** 1991-08

**Authors:** M. W. Lindegaard, N. Aass, E. S. Bue, L. Theodorsen, S. D. Fosså

**Affiliations:** Department of Nuclear Medicine, Norwegian Radium Hospital, Oslo.

## Abstract

Glomerular filtration rate (GFR), 131I-Hippuran clearance and estimated creatinine clearance were investigated in 34 patients with cancer. For Hippuran clearance and GFR, analysed with the X-ray contrast (iohexol) and fluorescence technique, the least square linear regression coefficient was 5.01 +/- 0.41 (r = 0.91). This value concurs with the five to one ratio between GFR and renal plasma flow known from normal physiology and supports that Hippuran clearance is a valid measure of renal function. When the individual values of Hippuran clearance were divided by 5.01, the mean difference between the methods was 0.4 ml min-1 1.73 m-2 with standard deviation 13.4 ml min-1 1.73 m-2. The lower and upper limits of agreement were -26.7 and 25.9 ml min-1 1.73 m-2, respectively. Comparing creatinine clearance estimated from the serum creatinine level with GFR, the limits of agreement were -29.4 and 21.6 ml min-1 1.73 m-2. These agreement limits are in the same range as those which can be calculated from the data from other studies.


Br. J. Cancer (1991), 64, 401-405 © Macmillan Press Ltd., 1991

### Glomerular filtration rate, $^{131}$I-Hippuran clearance and estimated creatinine clearance in cancer patients

M.W. Lindegaard[1], N. Aass[2], E.S. Bue[1], L. Theodorsen[3] & S.D. Fosså[2]

[1]*Department of Nuclear Medicine,* [2]*Department of Medical Oncology and Radiotherapy, and* [3]*The Central Laboratory; The Norwegian Radium Hospital, Oslo, Norway.*

**Summary** Glomerular filtration rate (GFR), $^{131}$I-Hippuran clearance and estimated creatinine clearance were investigated in 34 patients with cancer. For Hippuran clearance and GFR, analysed with the X-ray contrast (iohexol) and fluorescence technique, the least square linear regression coefficient was 5.01 ± 0.41 ($r = 0.91$). This value concurs with the five to one ratio between GFR and renal plasma flow known from normal physiology and supports that Hippuran clearance is a valid measure of renal function. When the individual values of Hippuran clearance were divided by 5.01, the mean difference between the methods was 0.4 ml min$^{-1}$ 1.73 m$^{-2}$ with standard deviation 13.4 ml min$^{-1}$ 1.73 m$^{-2}$. The lower and upper limits of agreement were − 26.7 and 25.9 ml min$^{-1}$ 1.73 m$^{-2}$, respectively. Comparing creatinine clearance estimated from the serum creatinine level with GFR, the limits of agreement were − 29.4 and 21.6 ml min$^{-1}$ 1.73 m$^{-2}$. These agreement limits are in the same range as those which can be calculated from the data from other studies.

Knowledge of the renal function is important in patients scheduled for potentially nephrotoxic treatment. Investigations allowing concurrent functional assessment and urinary tract imaging have a particular role in the management of patients with urogenital cancer in whom obstruction of the upper urothelial tract is a special risk. This goal is achieved with $^{131}$I-labelled Hippuran which has been the routine method for renal function studies in our department. The Hippuran clearance provides an estimate of glomerular filtration and tubular secretion (Ganong, 1977).

The glomerular filtration rate (GFR), however, seems to be the kidney function variable used most commonly in clinical oncology. GFR can be measured from the clearance of X-ray contrast media (Grönberg *et al.*, 1983; Sjöberg *et al.*, 1987; Effersöe *et al.*, 1990). Combining X-ray investigation using contrast media and GFR measurement seems attractive from a clinical point of view.

The present study was conducted in a series of 34 patients with cancer (33 with urogenital cancer). The study objective was to compare Hippuran clearance and creatinine clearance estimated from serum creatinine values with the GFR assessed with iohexol fluorescence technique.

### Patients and methods

Between November 1989 and April 1990, 35 consecutive cancer patients underwent investigations of glomerular filtration rate (GFR) using iohexol fluorescence technique, and Hippuran clearance. In one patient with testicular cancer, the blood samples for the Hippuran clearance measurement was contaminated with the radiotracer. This patient was excluded, leaving 34 patients for evaluation (Table I). All were normotensive without known glomerular disease and none had previously received cytotoxic therapy. None had serum creatinine levels >300 μmol l$^{-1}$ (upper limit 125 μmol l$^{-1}$), clinical signs of oedema, ascites, or pleural effusion, or known allergic disorder. The protocol included two blood samples for serum creatinine measurement, one obtained concurrently with the GFR investigation and one obtained previously, usually on the day of admission. The protocol was approved by the regional ethical committee in medical research. All patients gave informed consent to participate.

### *Serum creatinine*

This was analysed using the Jaffe reaction. The day to day coefficient of variation was <3.5% during the study. These data were used to estimate creatinine clearance using Cockroft's formula, modified for SI units and normalised to ml min$^{-1}$ 1.73 m$^{-2}$ body surface (Cockroft & Gault, 1976; Lott & Hayton, 1978). This formula reads:

$$\text{Creatinine clearance (ml min}^{-1}\,1.73\,\text{m}^{-2}) = \frac{(140 - \text{age}) \bullet 2.12 \bullet \text{weight} \bullet K}{\text{serum creatinine} \bullet \text{body surface}}$$

with serum creatinine in μmol l$^{-1}$, age in years, weight in kg, body surface in square metres. The constant K is 0.85 for women and 1.00 for men.

### *$^{131}$I-Hippuran clearance*

This was measured using the Oberhausen method (Oberhausen, 1977). The patients drank 500 ml of water before the investigation. To avoid vasovagal episodes during the 30 min of data acquisition, the patients were kept in the supine position. The blood pressure was measured repeatedly. No changes outside ± 10% of the initial value occurred. Five MBq $^{131}$I-Hippuran (Institutt for Energiteknikk, Kjeller, Norway) dissolved in 2 ml normal saline was injected in an indwelling cannula followed by 20 ml normal saline. Blood samples were taken from the same cannula 15 and 25 min after the injection. Renograms, split kidney function, and total Hippuran clearance were calculated by a manufacturer-supplied computer software (Siemens/Searle, Sonntag (1983)).

### *Glomerular filtration rate (GFR) using iohexol and fluorescence technique*

The injection of iohexol followed immediately after finishing the Hippuran study. The indwelling cannula was finally

**Table I** Summary of 34 patients with urogenital cancer

| Diagnosis | *No. (patients)* | *Age median (range)* |
|---|---|---|
| Bladder ca. | 14 (3)* | 73 (50-80) |
| Testicular ca. | 12 (–) | 39 (18-61) |
| Renal ca. | 4 (1) | 59.5 (58-73) |
| Prostatic ca. | 3 (–) | 67 (63-71) |
| Myelomatosis | 1 (–) | 59 |
| Total | 34 (4) | 62 (18-80) |

*Number of females in parenthesis.

Correspondence: M.W. Lindegaard, Department of Nuclear Medicine, The Norwegian Radium Hospital, Montebello, N-0310 Oslo 3, Norway.
Received 19 December 1990; and in revised form 25 March 1991.

flushed with 20 ml saline. The patients then returned to the ward. No food, fluid or smoking restrictions were issued. The individual iohexol dose (Omnipaque 350 mg iodine per ml, Nycomed, Oslo, Norway), ranged from 4 to 30 ml, and was determined from the patient's estimated creatinine clearance and body weight, using a nomogram supplied by the manufacturer.

Serum concentrations of iohexol were determined using an iodine fluorescence analyser (Renalyser PRX90, Provalid AB, Lund, Sweden). The method has been described elsewhere (Grönberg *et al.*, 1983; Sjöberg *et al.*, 1987). When the study started, no recommendation concerning the best sampling time for a one-point GFR determination was available. Samples were therefore drawn from the indwelling cannula after about 2, 3 and 4 h in patients with estimated creatinine clearance $< 100$ ml min$^{-1}$ 1.73 m$^{-2}$, and after about 1, 2 and 3 h in patients with estimated clearance $\geqslant 100$ ml min$^{-1}$ 1.73 m$^{-2}$. The samples were frozen and analysed in one batch at the end of the study when a software option providing a recommended sampling time for one-point analysis given the patient's serum creatinine, age, weight and height had become available. The sample obtained nearest to this recommended time was used for one point GFR estimation ($GFR_1$). Data from all three samples were used to obtain the GFR slope ($GFR_3$).

### *Data analysis*

The data are presented as means with standard errors (SEM), unless otherwise is stated. Commercially available microcomputer software was used. The mean difference (D) between selected variables and the standard deviation of the differences ($s_D$) were calculated. With differences approximately normally distributed, 95% of the differences will lie between D-1.96•$s_D$ and D + 1.96•$s_D$. These values, referred to as the 'limits of agreement', have standard errors of approximately $\sqrt{(3s_D^2 n^{-1})}$, where n is the sample size (Bland & Altman, 1986).

### Results

### *Glomerular filtration rate (GFR), iohexol fluorescence technique*

The GFR values obtained from the slope ($GFR_3$) ranged from 15 to 144 ml min$^{-1}$ 1.73 m$^{-2}$, mean: 76.4 ± 4.89 (± SEM). The values obtained from one-point estimation ($GFR_1$) ranged from 15 to 150 ml min$^{-1}$ 1.73 m$^{-2}$, mean 78.2 ± 4.93. The correlation coefficient from linear regression analysis was 0.98 (Table II). The mean difference between $GFR_1$ and $GFR_3$ was 1.7 ml min$^{-1}$ 1.73 m$^{-2}$, standard deviation 5.4 ml min$^{-1}$ 1.73 m$^{-2}$, giving lower and upper limits of agreement of − 8.8 and 12.2 ml min$^{-1}$ 1.73 m$^{-2}$, respectively (Table III).

**Table II** Renal function variables in 34 patients with urogenital cancer

| *Regression* | $a \pm SE_a$ | $b \pm SE_b$ | $s_{y.x}$ | *r* |
|---|---|---|---|---|
| $GFR_1$ on $GFR_3$ | 3.04 ± 2.63 | 0.98 ± 0.032 | 5.4 | 0.982 |
| $Cl_{est}$ on $GFR_3$ | 8.71 ± 5.94 | 0.83 ± 0.073 | 12.2 | 0.894 |
| $ClM_{hipp}$ on $GFR_3$ | 0.46 ± 6.62 | 1.00 ± 0.081 | 13.6 | 0.907 |
| $Cl_{prior}$ on $Cl_{est}$ | 1.60 ± 4.69 | 0.96 ± 0.061 | 9.5 | 0.940 |
| $ClM_{hipp}$ on $Cl_{est}$ | 1.46 ± 7.63 | 1.04 ± 0.100 | 15.5 | 0.879 |
| $ClM_{hipp}$ on $Cl_{prior}$ | 13.2 ± 9.9 | 0.89 ± 0.131 | 20.7 | 0.769 |

Least square linear regression; model: Y = (a ± $SE_a$) + (b ± $SE_b$)•X. $GFR_1$ and $GFR_3$: Glomerular filtration rate (GFR) investigation by iohexol fluorescence one-point analysis and slope, respectively. $Cl_{est}$: Creatinine clearance estimated from serum creatinine on day of GFR investigation. $Cl_{prior}$: Creatinine clearance from serum creatinine measured 1–3 days before GFR investigation. $Cl_{hipp}$: Hippuran clearance. $ClM_{hipp}$: $Cl_{hipp}$ divided by 5.01, see text. $s_{y.x}$: Square root of the mean square of residuals with 32 degrees of freedom denotes the amount of variability in the dependent variable (Y) not explained by the estimated model. All variables in ml min$^{-1}$ 1.73 m$^{-2}$.

**Table III** Comparison of renal function variables in 34 patients with urogenital cancer

| | | | *Limits of agreement* | | |
|---|---|---|---|---|---|
| *Variables* | *D* | $s_D$ | *Lower* | *Upper* | *95% CI* |
| $GFR_1$ on $GFR_3$ | 1.7 | 5.4 | − 8.8 | 12.2 | ± 1.6 |
| $Cl_{est}$ on $GFR_3$ | − 3.9 | 13.0 | − 29.4 | 21.6 | ± 7.8 |
| $ClM_{hipp}$ on $GFR_3$ | 0.4 | 13.4 | − 26.7 | 25.9 | ± 8.0 |
| $Cl_{prior}$ on $Cl_{est}$ | − 1.2 | 9.4 | − 19.7 | 17.3 | ± 5.6 |
| $Cl_{est}$ on $ClM_{hipp}$ | − 4.4 | 15.2 | − 34.3 | 25.5 | ± 9.0 |
| $Cl_{prior}$ on $ClM_{hipp}$ | − 5.3 | 20.6 | − 46.0 | 34.8 | ± 12.2 |

D: Mean of differences between variables. $s_D$: Standard deviation of D. Lower and upper limits of agreement (95% level): D ± 1.96•$s_D$•95% CI: 95% confidence interval for the agreement limit, 33 degrees of freedom. All variables in ml min$^{-1}$ 1.73 m$^{-2}$, see Table II for abbreviations.

### *Hippuran clearance*

The Hippuran clearance ($Cl_{hipp}$) was from 147 to 776 ml min$^{-1}$ 1.73 m$^{-2}$, mean 381 ± 27.4 ml min$^{-1}$ 1.73 m$^{-2}$. The best fit linear function by least square regression of $Cl_{hipp}$ on $GFR_3$ was: $Cl_{hipp}$ = (2.2 ± 33.1) + $GFR_3$• (5.01 ± 0.41). The square root of the mean square of residuals ($s_{y.x}$): with 32 degrees of freedom (d.f.) was 68.2 ml min$^{-1}$ 1.73 m$^{-2}$. This parameter denotes the amount of variability in the dependent variable ($Cl_{hipp}$) not explained by the estimated model. The correlation coefficient (r) was 0.907. To further test the relationship between $Cl_{hipp}$ and $GFR_3$, the series was divided in two equally large subseries, consisting of the patients with the 17 lowest and the 17 highest $GFR_3$ values, respectively. Analysis of variance (ANOVA) on the squared residuals from the regression analysis indicated no significant difference between the subseries (F-ratio 0.004, 1 d.f., $P > 0.95$). Thus, we found no evidence that the residuals were dependent on the absolute $GFR_3$ value. For the data from the 17 patients with $GFR_3$ values from 15 to 74 ml min$^{-1}$ 1.73 m$^{-2}$, the best fit linear function was:
$Cl_{hipp}$ = (28.1 ± 55.9) + $GFR_3$•(4.50 ± 1.03),
with $s_{y.x}$ = 64.9 ml min$^{-1}$ 1.73 m$^{-2}$, 15 d.f., $r = 0.746$.
The best fit linear function for the 17 patients with $GFR_3$ values from 76 to 144 ml min$^{-1}$ 1.73 m$^{-2}$ was:
$Cl_{hipp}$ = (− 27.8 ± 110.4) + $GFR_3$• (5.31 ± 1.09),
with $s_{y.x}$ = 74.8 ml min$^{-1}$ 1.73 m$^{-2}$, 15 d.f., $r = 0.781$ (Figure 1).

**Figure 1** For the 17 patients with $GFR_3$ values from 15 to 74 ml min$^{-1}$ 1.73 m$^{-2}$ (white dots) the regression line was $r = 0.746$, thin line, and for the 17 patients with $GFR_3$ values from 76 to 144 ml min$^{-1}$ 1.73 m$^{-2}$ (black dots) 0.781, bold line. These regression lines were not significantly different from each other or from the common regression line (dotted).

Although the $r$-value improved (from $<0.8$ to $>0.9$) when the data from all patients were combined, $s_{y.x}$ did not improve. This illustrates the value of considering the residuals when comparing data by means of regression analysis (Snedecor & Cochran, 1980). The $r$-value alone may give a false impression of consistency.

To assess the agreement between $GFR_3$ and $Cl_{hipp}$ and to allow other comparisons as well, the $Cl_{hipp}$ values were divided by 5.01 (the regression coefficient estimated for all 34 patients) to obtain the $ClM_{hipp}$ (Table II and Table III).

### *Estimated creatinine clearance*

The creatinine clearance estimated from the first blood sample ($Cl_{prior}$) ranged from 29 to $132\,ml\,min^{-1}\,1.73\,m^{-2}$, mean $70.5 \pm 4.73\,ml\,min^{-1}\,1.73\,m^{-2}$, and from 28 to 123 ml $min^{-1}\,1.73\,m^{-2}$, mean $71.8 \pm 4.63\,ml\,min^{-1}\,1.73\,m^{-2}$ for the second sample ($Cl_{est}$). The mean of differences, standard deviations and lower and upper limits of agreement are shown in Table III and Figure 2.

Creatinine clearance estimated concurrently with the GFR investigation ($Cl_{est}$) and previously ($Cl_{prior}$) was compared with GFR and Hippuran clearance. The results from linear regression analysis are listed in Table II. The mean of the differences, their standard deviations and the limits of agreement with 95% confidence intervals are demonstrated in Table III. A scatterplot of $Cl_{est}$ *vs* $GFR_3$ is shown in Figure 3a with limits of agreement in Figure 3b.

### Discussion

Smith and colleagues were the first to fully exploit the possibilities of clearance methods to quantify GFR and renal plasma flow (Smith *et al.*, 1949). Normally about 120 ml of filtrate are separated from the 600 ml of plasma passing through the kidneys each minute, which implies a plasma flow to GFR ratio of about 5 to 1 (de Wardener, 1985). The proportionality coefficient of $5.01 \pm 0.41$ between Hippuran clearance and GFR measured by iohexol fluorescence is close to this ratio and confirms the link between glomerular and tubular function embodied in the 'intact nephron hypothesis' (Bricker *et al.*, 1960). Since the extraction fraction for Hippuran is very high, 80–90%, the Hippuran clearance is often referred to as the 'effective renal plasma flow'. However, substances which compete with Hippuran for transport on

**Figure 2** Difference against mean for estimated creatinine clearance from serum creatinine obtained on different days ($Cl_{prior}$ and $Cl_{est}$). Lower and upper limits of agreement were $-19.7$ and $17.3\,ml\,min^{-1}\,1.73\,m^{-2}$, respectively, with 95% confidence interval (boxed area) of $\pm 5.6\,ml\,min^{-1}\,1.73\,m^{-2}$.

**Figure 3a** Scatterplot of glomerular filtration rate measured with the iohexol fluorescence technique ($GFR_3$) and creatinine clearance estimated from serum creatinine sampled at the same time ($Cl_{est}$). **b** difference against mean for estimated creatinine clearance and $GFR_3$, from **a**. Upper and lower limits of agreement $-29.4$ and $21.6\,ml\,min^{-1}\,1.73\,m^{-2}$ respectively, with 95% confidence intervals (boxed areas) of $\pm 7.8\,ml\,min^{-1}\,1.73\,m^{-2}$.

the tubular level also reduce the Hippuran extraction fraction (Maisey *et al.*, 1983). In the presence of such substances, for example cisplatin, the Hippuran clearance will underestimate kidney function. The GFR does not rely on tubular transport and is preferred under such circumstances. The one point measurement of iohexol clearance ($GFR_1$) agreed well with the slope ($GFR_3$). However, when using one cannula for injection and blood sampling, tracer contamination can be revealed only by using more than one sample.

The present study mutually compared GFR, Hippuran clearance and creatinine clearance estimated from serum creatinine values. The average error were close to zero (Table III). In some patients with discrepancy was nevertheless substantial and could have had clinical consequence. Variability in renal function studies may be due to methodological error. Inaccuracy in the measurement of injected and sampled tracer and X-ray contrast has been estimated to be responsible for a 4.5% difference for $^{51}$Cr-EDTA and 7.8% for X-ray contrast (Sjöberg *et al.*, 1987). Discrepancy may also arise from biological differences; in physical activity, in the intake of food and fluids, and from variations in blood pressure and renal blood flow. The impact of these factors is difficult to assess, and their minimisation probably requires strict patient regimens which may be hard to fulfill in clinical settings. In the present series some young patients with testicular cancer had higher serum creatinine levels in the first sample. It could be assumed that these patients had had a higher level of physical activity and/or a liberal intake of cooked meat before admission. Serum creatinine levels may increase substantially after eating cooked meat (Jacobson et al., 1979) and following exercise (Statland et al., 1973). We have observed serum creatinine levels rising transiently to more than twice the upper reference limit following generalised epileptic seizures in patients without kidney disease (unpublished data). Some elderly patients had low creatinine levels initially, perhaps reflecting poor nourishment before admission. This could explain why the discrepancy between the Hippuran clearance and the initial creatinine clearance estimation ($Cl_{prior}$) was greater than between the Hippuran clearance and the creatinine clearance estimated from blood sampled the same day ($Cl_{est}$), (Table III). For inpatients there is less variation in exercise and the composition of meals. We therefore suspect that the differences will be even greater in outpatients who are investigated at any time during the day.

Estimations of renal function imply the assumption of steady state conditions during the sampling period. The traditional reference method, the inulin clearance, has a standard deviation amounting from 5 to 7% of the mean when meticulous techniques are used (Davies & Shock, 1950). Comparing the precision and reproducibility of $^{51}$Cr-EDTA, estimated creatinine clearance and measured creatinine clearance, Bröchner-Mortensen et al. (1976) concluded that $^{51}$Cr-EDTA is the method of choice. However, the cyclotron-produced tracer $^{51}$Cr is less readily available than $^{99m}$Tc which also allows imaging of the urinary tract. GFR measurements using $^{99m}$Tc-DTPA is in widespread clinical use (Mulligan et al., 1990). In our institution six to eight blood samples during 3 h have been necessary to obtain a reliable result (unpublished data). Moreover, DTPA binding to plasma proteins may be a source of error (Russell et al., 1983).

O Reilly et al. (1986) correlated findings in 33 patients, under controlled conditions in a urology unit. The correlation coefficient between measured creatinine and $^{51}$Cr-EDTA clearances, was $r = 0.69$ (considered as unsatisfactory) while the correlation between $^{51}$Cr-EDTA and X-ray contrast medium clearances was $r = 0.90$ (good). However, the present results demonstrate that when two methods of clinical measurements are compared in terms of the correlation coefficient only ($r$, Table II), unrealistic impressions of consistency may result. It is more useful clinically to know how much the measurement with one method is likely to differ from that obtained with the other, as is expressed by the limits of agreement (Bland & Altman, 1986). This approach is relatively new, and we have therefore compared the limits of agreement from the present study (Table III) with those which can be calculated from other reports. Effersöe et al. (1990) correlated GFR by the iohexol method and the $^{51}$Cr-EDTA clearance in 15 patients and obtained $r = 0.95$. However, from their Table II the GFR values with the iohexol method were on the average 10.8 ml min$^{-1}$ 1.73 m$^{-2}$ higher (significantly greater than 0). The standard deviation of the differences was 7.9 ml min$^{-1}$ 1.73 m$^{-2}$, which implies lower and upper agreement limits of about $-5$ ml min$^{-1}$ 1.73 m$^{-2}$ and about 25 ml min$^{-1}$ 1.73 m$^{-2}$, respectively. Sjöberg et al. (1987) investigated 21 patients and compared GFR values obtained with metrizoate and $^{51}$Cr-EDTA. From their tabulated data (Sjöberg et al., 1987, Table I) the mean difference between the methods was approximately 3 ml min$^{-1}$ 1.73 m$^{-2}$, with standard deviation 10 ml min$^{-1}$ 1.73 m$^{-2}$. Hence, the limits of agreement were $-17$ ml min$^{-1}$ 1.73 m$^{-2}$ and 23 ml min$^{-1}$ 1.73 m$^{-2}$, respectively. Lewis et al. (1989) found $r = 0.86$ for X-ray contrast clearance and inulin clearance measurements. From their tabulated data (Lewis et al., 1989, Table I) the mean difference between the methods was 0.7 ml min$^{-1}$ 1.73 m$^{-2}$, with standard deviation 17.7 ml min$^{-1}$ 1.73 m$^{-2}$. This yields limits of agreement of about $-34$ and about 36 ml min$^{-1}$ 1.73 m$^{-2}$. Thus, the present limits of agreement are in the same range as those which can be calculated from the data from other studies. Our findings therefore seem to give a representative account of kidney function tests. To measure glomerular filtration rate within limits of less than $\pm$ 15–20 ml min$^{-1}$ 1.73 m$^{-2}$ seems not realistic, except, conceivably, under meticulously standardised regimens which may be difficult to implement in routine work. These considerations could be clinically important if nephrotoxic therapy guided by the individual patient's kidney function is contemplated.

### Conclusions

Creatinine clearance estimations based on the serum creatinine level is subject to the influence from the patient's muscle mass, physical activity and intake of cooked meat. The Hippuran clearance will underestimate kidney function in the presence of substances which compete with Hippuran for transport on the tubular level, for example cisplatin. The GFR method does not rely on tubular transport and is therefore the preferred method under such circumstances. Iohexol clearance with stable iodine and fluorescence technique is a cost effective mean to assess GFR. Using stable iodine allows almost infinite storage of standard solutions and patient samples. Thus, instrumentation and procedures can be controlled for quality and consistency when the need arises. This is difficult using a decaying radiotracer.

Author NA receives a research fellowship from The Norwegian Cancer Society.

The scientific assistance of Thomas Grönberg, Provalid AB, Lund, Sweden, is greatly appreciated.

Loan of the Renalyzer PRX90 instrument was provided by Provalid AB, Lund, Sweden.

### References

BLAND, J.M. & ALTMAN, D.G. (1986). Statistical methods for assessing agreement between two methods of clinical mesurements. *Lancet*, **i**, 307.

BRICKER, N.S., MORRIN, P.A.F. & KIME, S. (1960). The pathologic physiology of chronic Bright's disease. An exposition of the 'Intact Nephron Hypothesis'. *Amer. J. Med.*, **28**, 77.

BRÖCHNER-MORTENSEN, J. & RÖDBRO, P. (1976). Selection of routine method for determination of glomerular filtration rate in adult patients. *Scand. J. Clin. Lab. Invest.*, **36**, 35.

COCKROFT, D.W. & GAULT, M.H. (1976). Prediction of creatinine clearance from serum creatinine. *Nephron*, **16**, 31.

DAVIES, D.F. & SHOCK, N.W. (1950). The variability of measurement of inulin and diodrast tests of kidney function. *J. Clin. Invest.*, **29**, 491.

EFFERSÖE, H., ROSENKILDE, P., GROTH, S., JENSEN, L.I. & GOLMAN, K. (1990). Measurement of Renal Function with Iohexol. A comparison of Iohexol, $^{9m}$Tc-DTPA and $^{51}$Cr-EDTA clearance. *Invest. Radiol.*, **25**, 778.

GANONG, W.F. (1977). In *Review of Medical Physiology*. VIII. Formation and Excretion of Urine. p. 525. LANGE medical publications, Los Altos California.

GRÖNBERG, T., SJÖBERG, S., ALMEN, T., GOLMAN, K. & MATTSON, S. (1983). Noninvasive estimation of kidney function by X-ray fluorescence analysis. Elimination rate and clearance of contrast media injected for urography in man. *Invest. Radiol.*, **18**, 445.

JACOBSEN, F.K., CHRISTENSEN, C.K., MOGENSEN, C.E., ANDREASEN, F. & HEILSKOV, N.S.C. (1979). Pronounced increase in serum creatinine concentration after eating cooked meat. *Br. Med. J.*, **I**, 1049.

LEWIS, R., KERR, N., VAN BUREN, C. & 8 others (1989). Comparative evaluation of urographic contrast media, inulin, and $^{99m}$Tc-DTPA clearance methods for determination of glomerular filtration rate in clinical transplantation. *Transplantation*, **48**, 790.

LOTT, R.S. & HAYTON, W.L. (1978). Estimation of creatinine clearance from serum creatinine concentration – a review. *Drug. Intell. Clin. Pharm.*, **12**, 140.

MAISEY, M.N., BRITTON, K.E. & GILDAY, D.L. (1983). Clinical Nuclear Medicine. In *Renal Radionucleide Studies*. Britton, K.E. & Maisey, M.N. (eds) p. 124. Chapman and Hall: London.

OBERHAUSEN, E. (1977). Grundlagen der Nuklearmedizinischen Clearancebestimmung. In *Nuklearmedizinische Verfahren bei Erkrankungen der Nieren und ableitende Harnwege*. Pfannenstiel, P., Emrich, D., Oberhausen, E. & Pixberg, H.U. (eds) p. 21. Snetztor-Verlag: Konstanz.

O'REILLY, P.H., BROOMAN, P.J.C., MARTIN, P.J., POLLARD, A.J., FARAH, N.B. & MASON, G.C. (1986). Accuracy and reproducibility of a new contrast clearance method for the determination of glomerular filtration rate. *Br. Med. J.*, **293**, 234.

RUSSELL, C.D., BISCHOFF, P.G., ROWELL, K.L. & 4 others (1983). Quality control of Tc-99m DTPA for Measurement of glomerular filtration: Concise Communication. *J. Nucl. Med.*, **24**, 722.

SJÖBERG, S., HELLSTEN, S., ALMEN, T., GOLMAN, K. & GRÖNBERG, T. (1987). Estimating kidney function during urography. Comparison of contrast medium clearance and simultaneous $^{51}$Cr-EDTA clearance. *Acta Radiol.*, **28**, 587.

SMITH, H.W., GOLDRING, W. & CHASIS, H. (1949). The measurement of the tubular excretory mass, effective blood flow and filtration rate in the normal human kidney. *J. Clin. Invest.*, **17**, 263.

SNEDECOR, G.W. & COCHRAN, W.G. (1980). *Statistical Methods*, 7th edition. p. 149. Iowa State University Press, Ames, Iowa.

SONNTAG, A. (1983). Bestimmung der Nierenclearance durch Rekonstruktion der Ganzkörperretentionskurve aus späteren Messwerten. *Nucl. Med.*, **22**, 31.

STATLAND, B.E., WINKEL, P. & BOKELUND, H. (1973). Factors contributing to intra-individual variations of serum constituents: 2. Effects of exercise and diet on variation of serum constitutents in healthy subjects. *Clin. Chem.*, **19**, 1380.

DE WARDENER, H.E. (1985). Introduction to renal function and some theoretical considerations concerned in testing its integrity. In *The kidney, an outline of normal and abnormal function*. de Wardener, H.E. (ed.) p. 35. Churchill Livingstone: Edinburgh, London, New York.